# Diseases among Orang Asli community in Malaysia: a systematic review

**DOI:** 10.1186/s12889-022-14449-2

**Published:** 2022-11-16

**Authors:** Muhammad Hilmi Mahmud, Ummi Mirza Baharudin, Zaleha Md Isa

**Affiliations:** Department of Community Health, Faculty of Medicine, Universiti Kebangsaan Malaysia, Jalan Yaacob Latif, Bandar Tun Razak, 56000 Cheras, Kuala Lumpur, Malaysia

**Keywords:** Prevalence, Orang Asli, Peninsular Malaysia, Malnutrition, Soil-transmitted helminth

## Abstract

Orang Asli, the indigenous people of Peninsular Malaysia, is a minority population and placed behind in terms of socioeconomy, education and health. The Malaysian government has supported Orang Asli since the second Malaysia Plan in the 1970s, resulting in changes in their living standard, access to health, education and occupation. Hence, this review aims to assess the recent prevalence of diseases among Orang Asli in Peninsular Malaysia. The Preferred Reporting Items for Systematic Reviews and Meta-Analysis (PRISMA) review protocol guided this systematic review. The research question was formulated based on Population, Interest, Comparison and Outcome (PICO). The selected databases include Web of Science, Scopus, Ovid and EBSCOhost. The process of identification, screening and inclusion identified 33 articles. The mixed methods appraisal tool (MMAT) was then used to rank the quality of the articles. Selangor is the most frequent state involved in this review (12 studies), followed by Perak (10 studies), Pahang (10 studies) and Kedah (one study). We categorised into four categories of diseases, namely neglected tropical disease (*n* = 20), non-communicable disease (*n* = 6), nutritional status (*n* = 5), and hepatic disease (*n* = 2). The prevalence of diseases among Orang Asli is higher than in the general population, especially for soil-transmitted helminth (STH) and malnutrition. There is increasing research on non-communicable diseases due to an increasing trend in the socioeconomic status and lifestyle of the Orang Asli. Government agencies and private organisations must work together to empower Orang Asli with a healthy lifestyle, improve their socioeconomic status, and enhance food security, all of which will improve Orang Asli's health and reduce the prevalence of diseases.

**Supplementary Information:**

The online version contains supplementary material available at 10.1186/s12889-022-14449-2.

## Introduction

Indigenous peoples make up about 5% of the world's population, a small but dispersed group of people living all over the world [1]. An estimated 24 percent of the Earth's land surface is occupied by indigenous peoples who have acquired legal title to it [1]. In Malaysia, a combination of public data from the Department of Orang Asli Development (JAKOA) and data from the Malaysia Statistics Department estimated that the Indigenous Peoples of Malaysia made up 13.7 percent of the 32,382,300 million national population in 2018, including Orang Asli in Peninsular Malaysia and Natives from Sabah and Sarawak [2, 3].

Indigenous peoples in Peninsular Malaysia are known as Orang Asli. According to the Malaysian Law, Aboriginal Peoples Act 1954 (Act 134), Orang Asli can be defined as [4]: any person whose male parent is or was a member of an aboriginal ethnic group, who speaks an aboriginal language and habitually follows an aboriginal way of life and aboriginal customs and beliefs and includes a descendant through males of such persons.

In Peninsular Malaysia, there are three major tribal groups of Orang Asli: Negrito, Senoi, and Proto-Malay, each with its own culture, lifestyle and languages [5]. The three major tribal groups are further subdivided into 18 smaller sub-ethnic groups, which account for 1.1 percent of the peninsular population [2, 3]. Table 1 depicts the various tribes of Orang Asli in Peninsular Malaysia.

**Table 1:** Sub-ethnic group of three main Orang Asli tribal groups

Negrito	Senoi	Proto-Malay
Kensiu	Temiar	Temuan
Kintaq	Semai	Jakun
Jahai	Jah Hut	Semelai
Mendriq	Che Wong	Orang Kuala
Bateq	Semoq Beri	Orang Seletar
Lanoh	Mah Meri	Orang Kanaq

Source: The Department of Orang Asli Development [4]

According to the Department of Orang Asli Development (JAKOA) (2021), there were 178,197 Orang Asli in Peninsular Malaysia. Senoi is the largest tribe, accounting for 97,856 (54.9%), followed by Proto Malay 75,332 (42.3%) and the least is Negrito at 5,009 (2.8%). Most Orang Asli resides in Pahang (37.9%), followed by Perak (29.9%) [3]. No Orang Asli is recorded living in Perlis, Penang, and Wilayah Persekutuan [3].

Orang Asli is a minority population in Malaysia and is placed behind in socioeconomy, education, and health. Orang Asli community is known for their poor maternal health, iodine deficiency disorders, anemia, malnutrition, and intestinal parasitic infection [6]. The Malaysian government has supported the Orang Asli community since the second Malaysia Plan in the 1970s by increasing their educational standard, upgrading their basic facilities, improving their medical facilities and health status, and involving them in resettlement programme for Orang Asli [7]. Despite the Malaysian government's resettlement programme, only a few (0.7 percent) of Orang Asli were willing to move and live in the urban or semi-urban community, while the majority of them (62.0 percent) continue to live in rural and remote areas (37.0%) [7, 8]. Despite the Malaysian government's financial support for Orang Asli people in areas such as health, education, and employment opportunities, many tribes prefer to maintain their culture and traditional beliefs, which have a direct impact on people's health, behaviour toward diseases, and treatment adherence. Nevertheless, the Orang Asli community is undergoing lifestyle changes due to epidemiological and socioeconomic transitions. To our knowledge, even though Orang Asli are almost always excluded in the government national level survey, there are a lot of Orang Asli studies carried out over the past few years by university researchers investigating the effects of Orang Asli's epidemiological transition. Hence, this review is aimed to assess the prevalence of diseases among Orang Asli in Peninsular Malaysia.

## Methodology

### The review protocol

**Fig. 1:**
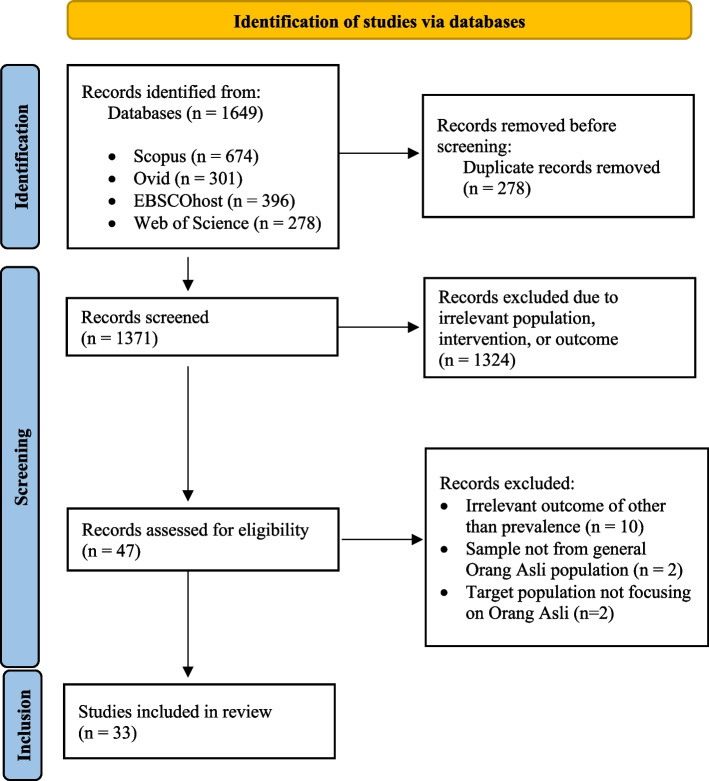
The PRISMA flow diagram [7]

This systematic review was guided by the Preferred Reporting Items for Systematic Reviews and Meta-Analysis (PRISMA) review protocol [9]. The authors began this review by conducting a systematic search into three stages: identification, screening, and inclusion, as shown in Fig. 1.

### Formulation of the research question

The research question for this study was developed using PICO. PICO is a tool that assists authors in developing a research question for the review. It is founded on the following fundamental ideas: The population or problem, the interest or intervention, the context or comparison, and the outcome [10]. Based on this, four main aspects were included in this review, namely Orang Asli (population), prevalence (interest), location (comparison) and diseases (outcome) which led the authors to the main objective of this review.

### Systematic searching strategies

The three main processes in systematic searching strategies are identification, screening, and inclusion, based on PRISMA review protocol (Fig. 1).

#### Identification

This step provides broader analysis of the related articles from the selected databases (Web of Science, Scopus, Ovid and EBSCOhost) for literature search that started from 3rd January 2022 and stopped at 13th January 2022. The identification process included looking for synonyms, Medical Subject Headings (MeSH) terms, related terms, and variations of the main keywords: Orang Asli, prevalence and diseases (Table 2). There were 1649 articles obtained from all the databases searched. A total of 272 duplicate articles were found and removed, leaving 1377 articles to be screened in the subsequent process (Fig. 1).

**Table 2:** Keywords used for systematic searching

Population	Interest	Outcome
Orang Asli	prevalence	diseases
indigenous	incidence	communicable disease
aboriginal	burden	acute
native	frequence	health
innate	chronic	nutrition
original	occurence	malnutrition
homegrown	epidemiology	infection
primitive	serological	parasitic
local	seroprevalence	infestation
ethnic		infectious
asal		gastroenteritis

#### Inclusion and exclusion

The screening process of the 1377 articles was carried out with the sorting function from each database. This review includes all articles that met the following criteria: original journal articles published in English between 2017 and 2021, observational or interventional studies with qualitative or quantitative data, outcome in terms of prevalence, and conducted among Orang Asli. We excluded systematic reviews, comments or letters to the editor, abstract conferences, animal studies, or in vivo or in vitro studies. This screening process include only 50 articles after exclusion of other articles due to irrelevant population, animal study, or outcome. Fifty full text articles were selected to determine eligibility by two reviewers. Only 33 articles were chosen in the final eligibility process with the rest excluded as they are not in line with the review objective (Fig. 1).

### Data extraction and analysis

The selected 33 articles were thoroughly read, paying particular attention to the abstract, method, results, and discussion. Then, the data were extracted from each article, including author, year of publication, study period, study type, study population, city and state, diseases studied, and prevalence results as tabulated in Additional file 1: Appendix A. After carefully identifying patterns in extracted data, the diseases were categorised accordingly. The categorised diseases were presented to a panel of expert of public health professionals who agreed that the categories were accurate and appropriate.

### Quality appraisal

The mixed methods appraisal tool (MMAT) was used to carefully examine how the data were extracted for analysis and validation [11]. Any disagreements were settled through discussion to reach a consensus. Finally, 33 articles were chosen for inclusion in the review. The final appraisal using MMAT is presented in Additional file 2: Appendix B.

## Results

This review included 33 articles in total. Most of the studies were published in 2020 (*n* = 13), followed by eight studies published in 2019, five published in both 2021 and 2018 each, and only two articles were published in 2017. Selangor is the most frequently mentioned state in this review, with 12 studies conducted in the state, followed by ten studies in Perak and ten studies in Pahang. The least is one study in Kedah, while three studies did not specify which state they were carried out. The sample size ranged from 58 to 1490 Orang Asli in the studies. One study used secondary data from the Malaysian Malaria Registry, comparing malaria cases among all ethnicities in Malaysia, [12] while the rest of the studies were exclusively carried out among Orang Asli community.

Interestingly, two studies compared Orang Asli communities living in resettlement areas to those living in remote forest areas [13, 14]. Cross-sectional study design has been utilised in all articles. This review categorize diseases into four, namely neglected tropical disease (*n* = 20), non-communicable disease (*n* = 6), nutritional status (*n* = 5) as well as hepatic disease (*n* = 3). Table 3 shows the qualitative evidence of the synthesis framework for the four themes of the disease classification among Orang Asli.

**Table 3:** Details of the four themes of the disease classification among Orang Asli

	**Article Code**																																	
**Disease Classification**	3	6	8	11	12	13	15	16	21	24	25	27	29	30	32	38	43	44	50	53	5	9	28	31	37	39	20	22	36	41	19	34	10	Total
**Neglected Tropical Disease**																																		
STH				/		/	/		/		/	/	/	/	/	/		/	/															12
Blastocystosis		/			/												/																	3
Giardiasis			/							/																								2
Amoebiasis								/												/														2
Malaria	/																																	1
**Non-communicable Disease**																																		
Hypertension																					/	/	/	/	/	/								6
Diabetes																					/	/	/	/	/	/								6
High triglyceridemia																					/	/		/										3
Hyper-cholesterolemia																					/		/			/								3
Low HDL																					/	/		/										3
**Nutritional Status**																																		
Underweight						/																			/			/		/				4
Stunting						/																												1
Wasting						/																												1
Overweight																					/				/			/	/	/				5
Obese																					/	/	/	/	/	/		/	/	/				9
Body fat analysis																														/				1
Vitamin D																											/							1
**Hepatic Disease**																																		
Hepatitis E virus																															/			1
Hepatitis B virus																																/		1
NAFLD																																	/	1

### Neglected tropical disease

Neglected tropical disease is the most frequent study found in this review of 20 studies. The majority of the studies involving soil-transmitted helminth (STH) amounted to 12 studies [13–24], followed by the protozoan type of parasite, which is blastocystosis involving three studies [25–27], giardiasis involving two studies [28, 29] and amoebiasis involving two studies [30, 31]. Lastly is one study involving malaria infection [12].

The reported prevalence of STH ranged from 14.1 to 98.4%. The highest prevalence recorded in this review was 98.4% in a study done among 122 Orang Asli from seven tribes in the states of Perak, Selangor, Johor and Pahang, where they used real-time PCR to diagnose STH compared to microscopy technique that can only detect 63.1% STH infection. [24] The lowest prevalence of STH infection was 14.1%, and the study involved 71 Orang Asli schoolchildren in Pos Lenjang, Pahang. [17] Most STH studies recorded more than 70% of STH infestation prevalence [13, 14, 16, 19–22, 24].

For blastocystosis, the prevalence ranged from 18.5 to 40.7%. The lowest prevalence came from a study done among relocated 58 Temiar Orang Asli in Gua Musang [26], while the highest prevalence came from a study done in Sungai Lembing, Pahang [27]. For amoebiasis, the highest recorded prevalence was 51.1% in a study done among schoolchildren in Kuala Kubu, Selangor [30], while another study recorded 26.3% prevalence among 411 Orang Asli in Selangor [31]. For malaria, Orang Asli recorded 550 cases (3.3%) from 16,500 cases for 2013–2017 [12].

### Nutritional status

Five studies reported the nutritional status among Orang Asli [14, 32–35]. The reported prevalence of underweight among Orang Asli children and adolescents was 59.1%, stunting was 45.8%, which was more prevalent among children in resettlement areas, while the reported prevalence of wasting was 42.3% [14]. The reported prevalence of anaemia among children and adolescents was 68.4% [14]. Among Orang Asli adult women, malnutrition was reported as 3.7%, obesity was prevalent in 26.2% of the population, and overweight was prevalent in 32.4% [33]. A study among Temiar Orang Asli adults showed that the prevalence of underweight was 9.0%, overweight was 28.0%, and obese was 23.0% [35]. The same study showed that 98.0% of adults had a normal nutritional status according to the measurement of the mid-upper-arm circumference (MUAC), while 69.0% of Orang Asli adults had a high and very high body fat analysis (BFA) [35]. The only study looking into vitamin D levels among Orang Asli of the Jah Hut tribe showed that 26.3% had a suboptimal serum 25-hydroxyvitamin D level where 24.9% were insufficient and 1.4% were deficient [32].

### Non-communicable disease

Six studies were conducted to determine the prevalence of metabolic syndrome among the Orang Asli population [32]. For abdominal obesity, the reported prevalence ranged from 15.9 to 72.6%, while the prevalence of hypertension ranged from 10.9 to 73.8%. Meanwhile, the prevalence of high triglyceride level ranged from 5.2 to 64.7%, while the prevalence of low high-density lipoprotein (HDL) cholesterol ranged from 46.6 to 59.7%. The prevalence of high fasting blood glucose and high HbA1C was 2.8 to 68.0% and 21.3 to 47.8%, respectively. The weighted mean prevalence for hypertension was 42.9%, high triglyceride 71.4%, low HDL 56.1% and diabetes mellitus 45.8%.

### Hepatic diseases

Only two studies investigated the prevalence of hepatitis E and B virus among Orang Asli. In a study among 207 participants from Temuan, Jah Hut and Mah Meri tribes, the prevalence of hepatitis E virus was 2.9% [36], whereas the prevalence of hepatitis B virus was 8.7% among 150 Negrito tribe participants [37]. Only one study looked at the prevalence of non-alcoholic fatty liver disease (NAFLD) in the Orang Asli community, and it discovered that the prevalence was 19.6%, with the age group 36–53 years old being the most affected [38] (Table 3).

## Discussion

This systematic review shows the current prevalence of diseases among Orang Asli. The findings showed that half of Orang Asli's research focused on parasitic infections, 11 studies focused on nutritional status, and only six focused on non-communicable diseases because Orang Asli is known for its poverty and still lives in remote rural areas far from public facilities, making them more vulnerable to parasitic infestations [39]. More than half of the studies were conducted in Selangor (*n* = 12), Perak (*n* = 11) and Pahang (*n* = 10) due to majority of Orang Asli communities (77.7%) live in these three states, which are located around central and west Peninsular Malaysia [40].

Although the prevalence range for STH infection was wide (14.1 to 98.4%), eight of 12 studies recorded STH infection as more than 70.0%. Interestingly, the remaining studies found STH prevalence rates ranging from 14.1 to 48.0% in primary schoolchildren [15, 17, 18, 23]. The prevalence is still much higher than in the general Malaysian population [41]. The lower prevalence among schoolchildren could be attributed to improved sanitation and hand washing facilities and the integration of health education and promotion in the classroom [15, 42]. Although effective intervention has been done to eliminate STH among Orang Asli, such as health education learning package (HELP), the disease is still dominant among Orang Asli due to a serious lack of personal hygiene, unimproved source of drinking water, and inadequate WASH facilities in school and residence [20, 43, 44].

Stunting is prevalent in up to 45.8 percent of Orang Asli children, much higher than the general population (21.8 percent) [14, 45]. This prevalence was even higher than in a 2019 Orang Asli study, which found that 19.2 percent of Orang Asli children aged 6 to 19 years were stunted [46]. The difference in prevalence may be contributed by the location of the studied area in which the Muslim et al. [13] study was carried out in a remote area while the Partap et al. [46] study was carried out in Segamat, which is considered a rural area. Stunting is also linked to untreated water, a lack of sanitation, and a high prevalence of STH among Orang Asli due to nutrient malabsorption caused by acute and chronic enteric infection [14, 41, 46]. Another factor of concern is the fact that the Orang Asli community is mainly a poor and low-income group with low purchasing power, faces challenges in farming and food searching activities, as well as having depleted food supply in their surrounding, which worsen food insecurity and further increase the risk of stunting [47, 48]. Enhanced food security and sufficient nutrient intakes among Orang Asli children should be made a vital agenda to improve undernourished problems [48]. In terms of overweight, Orang Asli has a lower prevalence than the national level at 30.4% since the majority of Orang Asli live in rural and remote areas that focus on farming and traditional food search, making them more physically active. Even though there was a report stating that Orang Asli, who lives in a semi-urban area, had an increased risk of being overweight due to increased intake of processed food and reduced physical activity [48–50]. For vitamin D level, Orang Asli adults showed 24.9% insufficiency and 1.4% deficiency which was much lower compared to the general Malaysian population [51]. This could be because Orang Asli's socioeconomic status and occupation forced them to be more exposed to sunlight [7, 52].

Non-communicable disease prevalence among Orang Asli, who live in the semi-urban area, was comparable to that of the general Malaysian population [50, 53, 54]. Non-communicable diseases have recently increased in prevalence in the Orang Asli population due to changes in nutrition and lifestyle habits [49]. Adapting to a modern lifestyle has changed dietary practices, social and lifestyle behaviours, leading to a more prevalent metabolic syndrome [55]. Continuous education and promotion regarding a healthy lifestyle should be given to the Orang Asli community to ensure they have good knowledge regarding risk factors of non-communicable diseases, translating into a better attitude and practice [56].

The first limitation of this review is that articles written in Malay are not included. The second limitation is that the retrieved studies were limited to those indexed in Web of Science, Scopus, Ovid and EBSCOhost. We also noted the imbalance of the selected articles, where half of the articles are regarding parasitic infestation. However, we believed our methodology follows the quality assessment standard, focusing on the Orang Asli population from multiple locations involving various indigenous tribes.

## Conclusion

We believed that this is the first comprehensive analysis of disease prevalence among the Orang Asli population in Malaysia. Numerous research were conducted among Orang Asli as a result of their socioeconomic and lifestyle changes, particularly in neglected tropical diseases, nutritional status, and non-communicable disease. In general, the prevalence of diseases among Orang Asli is higher than in the general population, particularly STH and malnutrition across all tribes and age categories. Government agencies and private organisations must work synergistically to educate Orang Asli about healthy lifestyle, socioeconomic uplift status, and enhance food security, all of which will improve Orang Asli's health and reduce the prevalence of diseases.

## Supplementary Information

**Additional file 1.** Appendix A.

**Additional file 2.** Appendix B. 

## Data Availability

All data generated or analysed during this study are included in this published article [and its supplementary information files].
