# Supplementary material for: Diseases among Orang Asli community in Malaysia: a systematic review
Source: BMC Public Health. 2022 Nov 16;22:2090. doi: 10.1186/s12889-022-14449-2 (PMC9670659; doi:10.1186/s12889-022-14449-2)
Supplement: Supplementary file 1 — Additional file 1. Appendix A. [file 12889_2022_14449_MOESM1_ESM.docx]

Appendix A

| **Article Code** | **Author, Year** | **Study period** | **Study type** | **Population study** | **City, State** | **Disease** | **Overall prevalence** | **Individual prevalence** |
| --- | --- | --- | --- | --- | --- | --- | --- | --- |
| **Neglected tropical diseases (20)** | | | | | | | | |
| 11 | Nisha & Davamani 2021 ^[24]^ | June to September 2 0 1 9 | Cross-sectional | n = 68; 35 females and 33 males | Hulu Langat, Selangor | Soil-transmitted Helminth (STH) Trichuris trichiura Ascaris lumbricoides | Total: 85 % |  |
| 13 | Muslim et al. 2021 ^[16]^ |  | Cross-sectional | Negritos N=343 (inland jungle=117, resettlement n=226) |  | STH | 292 (85.1%) | T. trichiura 244 (71.1%),  A. lumbricoides 159 (46.4%) Hookworm 68 (19.8%) |
| 15 | Othman et al. 2020 ^[26]^ | March 2012 and October 2013 | Cross-sectional | 122 aborigines from seven tribes Temiar, Jakun, Semai, Semelai, Mahmeri, Jahut, and Temuan | Perak, Selangor, Johor, and Pahang | STH | The real-time PCR 98.4% samples (n=122) Microscopy: 77 (63.1%) | Mono-helminth (microscopy) 42 (34.4); (PCR) 7 (5.7)  Poly-helminths 35 (28.7); 113 (92.6)  Two types 28 (23.0); 52 (42.6) Three types 7 (5.7); 51 (41.8) Four types 0 (0); 10 (8.2) |
| 21 | Nasr et al. 2020 ^[22]^ | January and April 2017 | Cross-sectional | 1,142 Orang Ali schoolchildren Senoi 560 (49.0) Proto-Malay 433 (37.9) Negrito 149 (13.0) | 1. Selangor (Hulu Selangor and Petaling) 2. Pahang (Raub and Lipis)  3. Negeri Sembilan (Jelebu and Kuala Pilah);  4. Kelantan (Gua Musang); 5. Johor (Segamat); 6. Perak (Hulu Perak, Muallim, Batang Padang) | STH | 70.1% found to be positive for at least one STH parasite species | A. lumbricoides 63.1% T. trichiura, 61.8%  hookworm infections 11.5% |
| 25 | Omran & Mohamed Kamel 2020 ^[25]^ |  | Cross-sectional | 60 Orang Asli (Temuan) primary school children | Dengkil, Selangor | STH | 38.30% | Trichuris trichiura 31.6% Ascaris lumbricoides 16.6%  Hookworm 11.6% Single species infection: 52.2%  Double infection: 30.4%  Triple infection: 17.4% |
| 27 | Aminuddin et al. 2020 ^[19]^ | January to March 2017 | Cross-sectional | 204 schoolchildren aged 7-17 years | Sungai Siput, Perak | STH | 48% (n= 97) | Monoparasitism (31%; n=63)  Moderate intensity by T trichuira (n=51, 25%). |
| 29 | Nisha et al. 2020 ^[23]^ | January to April 2017 | Cross-sectional | 190 villages | Hulu Langat, Selangor | STH | 136 (71.6%) | Trichuris trichiura: 127 (93.3%) Ascaris lumbricoides 65 (48.8%) |
| 30 | Adli et al. 2019 ^[17]^ |  | Cross-sectional | 71 volunteered aboriginal children Semai tribe aged between 1 to 12 years | Pos Lenjang, Pahang | Hookworm infestation | 14.1% |  |
| 32 | Al-Mekhlafi et al. 2019 ^[18]^ | January to April 2017 | Cross-sectional | 1,142 Orang Asli primary schoolchildren | Selangor, Pahang, Negeri Sembilan, Kelantan, Johor and Perak | STH strongyloidiasis | 15.80% |  |
| 38 | Muslim et al. 2019 ^[15]^ | May 2016 to April 2017 | Cross sectional | 416 Negrito participants grouped into two; Inland Jungle Villages (IJV); and Resettlement Plan Scheme (RPS) | Northern states of Peninsular Malaysia | STH | Overall any infection 362 (87.0%) Overall any STH infection 358 (86.1%) Overall any protozoa infection 106 (25.5%)  Prevalence of STH was significantly higher in IJV (91.3%) versus RPS (83.1%) | By species (STH): T. trichiura 300 (72.1) A. lumbricoides 180 (43.3) Hookworm 89 (21.4) Strongyloides sp. 7 (1.7)  By species (Protozoa): Entamoeba/Dientamoeba sp. 91 (21.9) Blastocystis sp. 18 (4.3) Giardia sp. 16 (3.8) Cryptosporidium sp. 12 (2.9) Other parasites (Flukes, Tapeworms, unidentified cyst and eggs) 37 (8.9)  Severe intensity of Trichuris trichiura infections higher in the RPS (17.2%) compared to IJV (6.5%) Severe Ascaris lumbricoides infection was observed at 20.0% amongst RPS Negritos and 15.0% amongst IJV hookworm infection, both prevalence and individuals with moderate to severe infections were higher in the IJV (26.2%, 41.0%) versus RPS |
| 44 | Mohd-Shaharuddin et al. 2018 ^[21]^ | October 2014 to May 2015 | Cross sectional | 411 Temuan indigenous subgroup | Selangor (Pangsun, Gurney, Hulu Tamu, Tun Razak, Kemensah | Soil-transmitted helminths (STH) | 72.70% | Trichuris trichiura: 58.4%, Ascaris lumbricoides: 45.5% Hookworm (23.1% |
| 50 | Brandon-Mong et al. 2017 ^[20]^ | October 2013 to April 2014 | Cross sectional | 235 samples | 1. Selangor (Gombak, Hulu Langat, Hulu Selangor, Klang, Kuala Langat, Sepang) 2. Kedah | Soil-transmitted helminths (STH) | 81.70% | T.trichiura (76.6%) Hookworms (26.4%)  A. lumbricoides (19.1%) |
| 6 | Mohammad et al. 2017 ^[30]^ | February to March 2015 | Cross-sectional | 253 participants  1 - 85 years old 187 (73.9%) Senois and 66 (26.1%) Proto-Malays | Sungai Lembing, Pahang | Blastocystis | 40.7% (103/253) Senois 43.9% Proto-Malays 31.8% | Aged >15 years had significantly highest prevalence (48.3%)  Children aged <15 years had the lowest prevalence (30.6%) |
| 12 | Mohamed Kamel & Najah 2021 ^[28]^ |  | Cross-sectional | 307 Orang Asli (Semai) schoolchildren  aged 6-14 years  (156 Males and 151 females | Pos Senderut, Pahang | Blastocystosis | Total: 21.2% | B. hominis  Male 25 (16.0)  Female 40 (26.5)  Total 65 (21.2) |
| 43 | Mohammad et al. 2018 ^[29]^ |  | Cross sectional | 58 respondents the relocated Temiar Orang Asli community | Gua Musang, Kelantan | Blastocytosis | The prevalence of Blastocystis infection by PCR assay was 18.5% (45/243) |  |
| 8 | Jeyaprakasam & Ghani 2019 ^[32]^ |  | Cross-sectional | 139 participants | Sungai Raba Village Gerik, Perak | Giardiasis | Total: 15.1%.  Males 24.2% Female 6.8% |  |
| 24 | Adli & Ghani 2020b ^[31]^ | Apr-15 | Cross-sectional | 208 Orang Asli primary school children | Pos Senderut, Pahang | Giardia intestinalis (protozoan parasite) - giardiasis | Prevalence of giardiasis was 34.6% |  |
| 16 | Adli & Ghani 2020a ^[33]^ | Apr-17 | Cross-sectional | n=92 schoolchildren (49 boys and 43 girls) | Kuala Kubu Bharu, Selangor | Amoebiasis | 51.10% | The highest infection rate aged between 7 < 10 years (55.3%) Males (57.1%) have a higher rate of infection vs females (44.2%) |
| 53 | Ngui et al. 2020 ^[34]^ | October 2014 to May 2015 | Cross sectional | 411 Orang Asli | Selangor (Gurney, Pangsun, Kemensah, Ulu Tamu, Tun Razak and Kuala Kubu Bharu) | Entamoeba | Overall occurrence of Entamoeba species of 26.3% (108/411) | Single infection of E. dispar (26.5%; 13/49) E. histolytica 20.4%  E. moshkovskii, 20.4%   Double infection of  E. dispar +E. moshkovskii: 10.2%,  E. dispar +E. histolytica: (8.2%)   E.moshkovskii and E. histolytica (6.1%).  Triple infection: 8.2% |
| 3 | Hussin et al. 2020 ^[14]^ | 2013-2017 (5 years) | Cross-sectional | 16,500 cases indigenous: 3.3% (n=550) | Whole Malaysia | Malaria | Indigenous: 3.3% (n=550) |  |
| **Non-communicable diseases (6)** | | | | | | | | |
| 5 | Ahmad et al. 2018 ^[11]^ | March 2013 to December 2013 | Cross-sectional | 274 participants 133 Orang Asli 141 Malays 93 males and 181 females < 45 years; > 45 years | Hulu Perak and Batang Padang, Perak | Cardiovascular risk factor Abdominal obesity Hypertension Abnormal glycaemia Dyslipidemia |  | < 45 years of age; >45 years of age Overweight and obesity: 43(41.3); 8(27.6) Abdominal obesity: 17.9%, 17.2% (n=17,15) Hypertension: 10.9%, 53.6% (n=11,15) Newly diagnosed DM/IFG: 1.9%, 6.9% (n=2,2) Hypercholesterolemia: 34.6%, 65.5% (n=36, 19) High LDL: 33(31.7); 18(62.1) Low HDL: 48(46.6);17(58.6) High TG: 22(21.4);13(44.8) |
| 9 | Sugathan et al. 2021 ^[13]^ | 2014 to 2016 | Cross-sectional | 145 participants | Gerik and Batang Padang, Perak | Metabolic Syndrome | Total: 20 (13.8%) Male 9.8% Female 15.4% | Abdominal obesity: 23 (15.9%) Hypertension: 50 (34.5%) Fasting Glucose: 4 (2.8%) Triglyceride: 36 (24.8%) HDL Cholesterol: 68 (46.9%) |
| 28 | Ithnin et al. 2020 ^[12]^ | November 2017 to December 2018 | Cross-sectional | 325 OA Temuan, the Proto-Malay | Jelebu & Kuala Pilah, Negeri Sembilan | Metabolic Syndrome |  | Hypertension 4 (14.8%),  Hypercholesterolemia 17(5.2%) Diabetes mellitus 14(4.3%) Abdominal obese 194 (59.7%)  Increased blood pressure 97 (29.8%) Increased blood glucose 18 (5.5) |
| 31 | Aghakhanian et al. 2019 ^[39]^ | Between 2010 and 2016 | Cross-sectional | (n = 629) from three major groups (Negrito, Proto-Malay, and Senoi | Perak Kelantan Pahang Johor Selangor Perak | Metabolic Syndrome | 29.57% | Central obesity (72.6)  BMI obesity (52.1)  High FBS 428 (68.0)  High HbA1c (21.3) High TG 407 (64.7)  Low HDL-C 376 (59.7) Hypertension (73.8) High UA (34.6) |
| 37 | Yeo et al. 2019 ^[41]^ |  | Cross sectional | Semi-urbanized 72 Temiar tribe | Kampong Pos Piah, Perak | Metabolic syndrome |  | Underweight (<18.5): 7 (10.3%) Overweight/obese: 41 (60.4%) HbA1C level:  Pre-diabetes (5.7-6.4%): 31 (44.9%), Diabetes: 2 (2.9%) Blood pressure Pre-hypertensive 39 (56.5%) Stage 1: 12 (17.4%) Stage 2: 4 (5.8%) |
| 39 | Wong et al. 2018 ^[40]^ | 2015 | Cross sectional | 72 Jakun Orang Asli Tasik Chini | Tasik Chini, Pekan, Pahang | Non- Communicable Diseases |  | 41.7% for hypertension,  25.0% for Diabetes mellitus,  6.9% for dyslipidaemia WC (cm)  Central obesity n=24, 33.3% > 80cm female > 90cm male |
| **Nutritional status (6)** | | | | | | | | |
| 13 | Muslim et al. 2021 ^[16]^ |  | Cross-sectional | Negrito's children and adolescents N=343 (inland jungle=117, resettlement n=226) |  | Malnutrition, STH | Malnutrition (RPS=74.3%, IJV 68.4%) Stunting 45.8% Wasting 42.3% Underweight 59.1%,  Anemia 68.4%. RPS 69.7% vs IJV 65.6% | Stunting is more critical in the RPS  Stunting (HAZ scores < -2SD) 30.2%(RPS) vs 33.6% (IJV) Severe stunting (HAZ scores < -3SD) were 18.1% (RPS) vs 7.3% (IJV) |
| 20 | Yin Chua et al. 2020 ^[38]^ |  | Cross-sectional | 555 (164 men, 391 women) Orang Asli adults aged 18–65 years of Jah Hut sub-tribe | Krau Wildlife Reserve (KWR) | Serum 25(OH)D (vitamin D) | Prevalence of suboptimal 25(OH)D concentration was 26.3% | 24.9% insufficiency (50 to <75 nmol/L) and  1.4% deficiency (<50 nmol/L) |
| 22 | Law et al. 2020 ^[35]^ |  | Cross-sectional | 355 Senoi Orang Asli women | Batang Padang, Perak | Nutritional status |  | Underweight 3.7% Overweight 32.4% Obesity 26.2% Total energy intake 1935 ± 534 Kcal |
| 36 | Yin et al. 2019 ^[37]^ | 2011-2012 and 2015-2016 surveys | Cross sectional | N2011-2012=828; N2015-2016=662 (total= 1490) Follow-up data were available for 378 adults (male:113; female:265) | Krau Wildlife Reserve | Overweight and obesity | 2011-2012: 26.2% 2015-2016: 35.6% | The prevalence of overweight and obesity  2011-2012: 18.8% and 7.4%  2015-2016: 26.1% and 9.5%   More than one-third (35.5%) of the adults had weight gain of more than 5.0% |
| 41 | Rohin et al. 2018 ^[36]^ |  | Cross sectional | 58 Temiar Orang Asli community | RPS Kuala Betis, Gua Musang, Kelantan | Non- Communicable Diseases |  | 9% underweight 28% overweight and 23% obese 16% were at risk of comorbidities and increased abdominal fat Based on MUAC, 98% of the respondents had normal nutrition. However, 41% had very high, 28% had high, 29% had normal and 2% had low body fat analysis (BFA) levels |
| **Hepatic Diseases (3)** | | | | | | | | |
| 19 | Wong et al. 2020 ^[42]^ | April 2011 to February 2013 Semi-structured interviews and observations: 17 October 2018 | Cross-sectional | 207 samples Temuan 180 (87.0%) Jah Hut 11 (5.3%) Mah Meri 16 (7.7%) | Jelebu, Negeri Sembilan Hulu Langat, Carey Island Selangor Temerloh, Pahang | Hepatitis E | 6 (2.9%) were positive for anti-HEV IgG None of the samples were positive for anti-HEV IgM | 5.9% were positive for anti-HEV IgG in samples from the village of Dusun Kubur |
| 34 | Sahlan et al. 2019 ^[43]^ |  | Cross-sectional | 150 participants from the Bateq (49.3%) and Mendriq (29.4%) sub-tribes | Five Negrito settlements in Kelantan and Perak | Hepatitis B virus | Hep B Virus 8.7% (13) |  |
| 10 | Abdul et al. 2021 ^[44]^ | January 2014-February 2016 | Cross-sectional | 270 participants |  | Non-alcoholic fatty liver disease (NAFLD) | Total: 19.6% | 83% moderate grade of fatty liver 17.0% mild grade 18-35 years 6.3% 36-53 years 28.6% 54-71 years 21.3% ≥ 72 years 0% |
