# Supplementary material for: Diseases among Orang Asli community in Malaysia: a systematic review
Source: BMC Public Health. 2022 Nov 16;22:2090. doi: 10.1186/s12889-022-14449-2 (PMC9670659; doi:10.1186/s12889-022-14449-2)
Supplement: Supplementary file 2 — Additional file 2. Appendix B. [file 12889_2022_14449_MOESM2_ESM.docx]

Appendix B

| **Article Code** | **Title** | MMAT METHODOLOGICAL QUALITY DATA | | | | | | | | | **Overall comment reviewer** | **Result (recommended- Y/N)** |
| --- | --- | --- | --- | --- | --- | --- | --- | --- | --- | --- | --- | --- |
|  |  |  |  | **S1** | **S2** | **Criteria according to study design type (refer MMAT 2018)** | | | | |  |  |
|  |  | **Category of study design (refer MMAT flow)** | **Code MMAT** | **Are there clear research question?** | **Do the collected data allow to address the research questions?** | **1.1/ 2.1/ 3.1/ 4.1/ 5.1** | **1.2/ 2.2/ 3.2/ 4.2/ 5.2** | **1.3/ 2.3/ 3.3/ 4.3/ 5.3** | **1.4/ 2.4/ 3.4/ 4.4/ 5.4** | **1.5/ 2.5/ 3.5/ 4.5/ 5.5** |  |  |
| 5 | Cardiovascular risk factors in rural Malays and Aborigines in Perak, Malaysia; An alarming situation | Cross-sectional | 4 | Yes | Yes | Yes | Yes | Yes | Yes | Yes |  | Yes |
| 9 | Socio demographic and lifestyle factors of metabolic syndrome among adult rural indigenous Malaysian population from Perak state, Malaysia | Cross-sectional | 4 | Yes | Yes | Yes | Yes | Yes | Yes | Yes |  | Yes |
| 22 | Predictors of overweight and obesity and its consequences among Senoi Orang Asli (indigenous people) women in Perak, Malaysia | Cross-sectional | 4 | Yes | Yes | Yes | Yes | Yes | Yes | Yes |  | Yes |
| 28 | Knowledge, attitude, and practices of non-communicable diseases: comparison between Orang Asli and Malay from rural area in Negeri Sembilan, Malaysia: a comparative study | Cross-sectional | 4 | Yes | Yes | Yes | Yes | Yes | Yes | Yes |  | Yes |
| 31 | Metabolic syndrome and cardiometabolic risk factors among indigenous Malaysians | Cross-sectional | 4 | Yes | Yes | Yes | Yes | Yes | Yes | Yes |  | Yes |
| 36 | Overweight and obesity among Orang Asli adults in Krau Wildlife Reserve, Pahang: a four-year follow-up study | Cross-sectional | 4 | Yes | Yes | Yes | Yes | Yes | Yes | Yes |  | Yes |
| 37 | Health and saliva microbiomes of a semi-urbanized indigenous tribe in Peninsular Malaysia | Cross-sectional | 4 | Yes | Yes | Yes | Yes | Yes | Yes | Yes |  | Yes |
| 39 | Prevalence and modifiable risk factors of non-communicable diseases among Jakun Orang Asli at Tasik Chini, Pekan, Pahang. | Cross-sectional | 4 | Yes | Yes | Yes | Yes | Yes | Yes | Yes |  | Yes |
| 41 | Nutritional status of the Temiar Orang Asli community in Kuala Betis, Gua Musang, Kelantan | Cross-sectional | 4 | Yes | Yes | Yes | Yes | Yes | Yes | Yes |  | Yes |
| 19 | The study of seroprevalence of hepatitis E virus and an investigation into the lifestyle behaviours of the aborigines in Malaysia | Cross-sectional | 4 | Yes | Yes | Yes | Yes | Yes | Yes | Yes |  | Yes |
| 34 | Hepatitis B virus infection: Epidemiology and seroprevalence rate amongst Negrito tribe in Malaysia | Cross-sectional | 4 | Yes | Yes | Yes | Yes | Yes | Yes | Yes |  | Yes |
| 3 | Updates on malaria incidence and profile in Malaysia from 2013 to 2017 | Cross-sectional | 4 | Yes | Yes | Yes | Yes | Yes | Yes | Yes |  | Yes |
| 6 | Prevalence and risk factors of Blastocystis infection among underprivileged communities in rural Malaysia | Cross-sectional | 4 | Yes | Yes | Yes | Yes | Yes | Yes | Yes |  | Yes |
| 8 | Giardiasis amongst the aboriginal school children at Sungai Raba village Gerik, Perak, Malaysia | Cross-sectional | 4 | Yes | Yes | Yes | Yes | Yes | Yes | Yes |  | Yes |
| 11 | Efficacy of single dose albendazole treatment of soil transmitted helminths among indigenous children in Malaysia | Cross-sectional | 4 | Yes | Yes | Yes | Yes | Yes | Yes | Yes |  | Yes |
| 12 | Blastocystosis amongst the Orang Asli (aborigine) schoolchildren at Pos Senderut, Pahang, Malaysia | Cross-sectional | 4 | Yes | Yes | Yes | Yes | Yes | Yes | Yes |  | Yes |
| 13 | Nutritional status, hemoglobin level and their associations with soil-transmitted helminth infections between Negritos (indigenous) from the inland jungle village and resettlement at town peripheries | Cross-sectional | 4 | Yes | Yes | Yes | Yes | Yes | Yes | Yes |  | Yes |
| 15 | Multiplex real-time PCR revealed very high prevalence of soil-transmitted helminth infections among aborigines in Peninsular Malaysia | Cross-sectional | 4 | Yes | Yes | Yes | Yes | Yes | Yes | Yes |  | Yes |
| 16 | Amoebiasis amongst the Orang Asli (aborigine) school children at Kuala Kubu Bharu, Selangor, Malaysia | Cross-sectional | 4 | Yes | Yes | Yes | Yes | Yes | Yes | Yes |  | Yes |
| 21 | A holistic approach is needed to control the perpetual burden of soil-transmitted helminth infections among indigenous schoolchildren in Malaysia | Cross-sectional | 4 | Yes | Yes | Yes | Yes | Yes | Yes | Yes |  | Yes |
| 24 | Giardiasis amongst the Orang Asli (aborigine) school children at Pos Senderut, Pahang, Malaysia | Cross-sectional | 4 | Yes | Yes | Yes | Yes | Yes | Yes | Yes |  | Yes |
| 25 | Soil-transmitted helminthiases amongst the Orang Asli (aboriginal) school children at a semi urban setting, Dengkil, Malaysia | Cross-sectional | 4 | Yes | Yes | Yes | Yes | Yes | Yes | Yes |  | Yes |
| 27 | Boarding school: a simple approach to reduce soil transmitted helminth infections in Orang Asli children of Sungai Siput, Perak, 2017 | Cross-sectional | 4 | Yes | Yes | Yes | Yes | Yes | Yes | Yes |  | Yes |
| 29 | Risk factors associated with soil transmitted helminth (STH) infection in two indigenous communities in Malaysia | Cross-sectional | 4 | Yes | Yes | Yes | Yes | Yes | Yes | Yes |  | Yes |
| 30 | Hookworm infestation amongst the Orang Asli (aboriginal) children at Pos Lenjang, Pahang, Malaysia | Cross-sectional | 4 | Yes | Yes | Yes | Yes | Yes | Yes | Yes |  | Yes |
| 32 | Prevalence and risk factors of *Strongyloides stercoralis* infection among Orang Asli schoolchildren: new insights into the epidemiology, transmission and diagnosis of strongyloidiasis in Malaysia | Cross-sectional | 4 | Yes | Yes | Yes | Yes | Yes | Yes | Yes |  | Yes |
| 38 | Prevalence, intensity and associated risk factors of soil transmitted helminth infections: A comparison between Negritos (indigenous) in inland jungle and those in resettlement at town peripheries | Cross-sectional | 4 | Yes | Yes | Yes | Yes | Yes | Yes | Yes |  | Yes |
| 43 | Subtype distribution of Blastocystis isolated from humans and associated animals in an indigenous community with poor hygiene in Peninsular Malaysia | Cross-sectional | 4 | Yes | Yes | Yes | Yes | Yes | Yes | Yes |  | Yes |
| 44 | Soil-transmitted helminthiasis among indigenous communities in Malaysia: Is this the endless malady with no solution? | Cross-sectional | 4 | Yes | Yes | Yes | Yes | Yes | Yes | Yes |  | Yes |
| 50 | Soil-transmitted helminths in Malaysia landscape: an aborigines study | Cross-sectional | 4 | Yes | Yes | Yes | Yes | Yes | Yes | Yes |  | Yes |
| 53 | Copro-molecular study of Entamoeba infection among the indigenous community in Malaysia: A first report on the species-specific prevalence of Entamoeba in dogs | Cross-sectional | 4 | Yes | Yes | Yes | Yes | Yes | Yes | Yes |  | Yes |
| 20 | Associations of serum 25-hydroxyvitamin D with adiposity and at-risk lipid profile differ for indigenous (Orang Asli) male and female adults of Peninsular Malaysia | Cross-sectional | 4 | Yes | Yes | Yes | Yes | Yes | Yes | Yes |  | Yes |
| 10 | Prevalence of ultrasound-diagnosed non-alcoholic fatty liver disease among rural indigenous population in Malaysian and its association with biochemical and anthropometric measures | Cross-sectional | 4 | Yes | Yes | Yes | Yes | Yes | Yes | Yes |  | Yes |
